# iNR-PhysChem: A Sequence-Based Predictor for Identifying Nuclear Receptors and Their Subfamilies via Physical-Chemical Property Matrix

**DOI:** 10.1371/journal.pone.0030869

**Published:** 2012-02-21

**Authors:** Xuan Xiao, Pu Wang, Kuo-Chen Chou

**Affiliations:** 1 Computer Department, Jing-De-Zhen Ceramic Institute, Jing-De-Zhen, China; 2 Gordon Life Science Institute, San Diego, California, United States of America; University of South Florida College of Medicine, United States of America

## Abstract

Nuclear receptors (NRs) form a family of ligand-activated transcription factors that regulate a wide variety of biological processes, such as homeostasis, reproduction, development, and metabolism. Human genome contains 48 genes encoding NRs. These receptors have become one of the most important targets for therapeutic drug development. According to their different action mechanisms or functions, NRs have been classified into seven subfamilies. With the avalanche of protein sequences generated in the postgenomic age, we are facing the following challenging problems. Given an uncharacterized protein sequence, how can we identify whether it is a nuclear receptor? If it is, what subfamily it belongs to? To address these problems, we developed a predictor called **iNR-PhysChem** in which the protein samples were expressed by a novel mode of pseudo amino acid composition (PseAAC) whose components were derived from a physical-chemical matrix via a series of auto-covariance and cross-covariance transformations. It was observed that the overall success rate achieved by **iNR-PhysChem** was over 98% in identifying NRs or non-NRs, and over 92% in identifying NRs among the following seven subfamilies: NR1
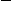
thyroid hormone like, NR2
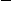
HNF4-like, NR3
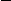
estrogen like, NR4
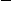
nerve growth factor IB-like, NR5
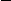
fushi tarazu-F1 like, NR6
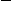
germ cell nuclear factor like, and NR0
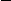
knirps like. These rates were derived by the jackknife tests on a stringent benchmark dataset in which none of protein sequences included has 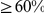 pairwise sequence identity to any other in a same subset. As a user-friendly web-server, **iNR-PhysChem** is freely accessible to the public at either http://www.jci-bioinfo.cn/iNR-PhysChem or http://icpr.jci.edu.cn/bioinfo/iNR-PhysChem. Also a step-by-step guide is provided on how to use the web-server to get the desired results without the need to follow the complicated mathematics involved in developing the predictor. It is anticipated that **iNR-PhysChem** may become a useful high throughput tool for both basic research and drug design.

## Introduction

Found within cells, nuclear receptors (NRs) are a class of proteins responsible for sensing steroid and thyroid hormones and certain other molecules. In response, these receptors work with other proteins to regulate the expression of specific genes, thereby controlling the development, homeostasis, and metabolism of the organism. A unique property of NRs that distinguishes themselves from other classes of receptors is their ability to directly interact with and control the expression of genomic DNA, and hence they are also classified as transcription factors [1], [2]. Since NRs bind small molecules that can be easily modified by chemical manipulation, and also since NRs control the functions closely associated with major diseases (such as cancer, osteoporosis, and diabetes), they have become promising pharmacological targets [3], [4], [5].

Grouped into a superfamily that includes receptors for steroid hormones, vitamin D, ecdysone, retinoic acid and thyroid hormone [6], [7], NRs are modular proteins composed of six distinct regions (A–F) [8], [9] that correspond to functional and structural domains. Not all the NRs contain all the six domains. Regions C and E display the highest degree of conservation. C is involved in DNA binding and E involved in ligand binding and dimerization. Owing to its high conservation, the C domain is the signature motif of the superfamily. It is composed of two zinc fingers; the presence of such feature facilitates the identification of NRs [5]. Based on the alignments of the conserved domains [4], [10], the superfamily has been subdivided into seven subfamilies [11], [12].

The importance of NRs has prompted a rapid accumulation of the relevant data from a great diversity of fields of research: sequences, expression patterns, 3-D (three-dimensional) structures, protein-protein interactions, target genes, physiological roles, mutations, etc. These accumulated data are very helpful for data mining and knowledge discovery. Since the function of a NR is closely correlated with which subfamily it belongs to, facing the avalanche of protein sequences generated in the post-genomic age, it is highly desired to develop automated methods for rapidly and effectively identifying NRs and their subfamilies according to their sequences information alone, because the knowledge thus acquired may benefit both basic research and drug development. Actually, some efforts have already been made in this regard.

In 2004, Bhasin and Raghava [13] have proposed a method for predicting the subfamilies of NRs using SVM as the prediction engine and the amino acid composition and dipeptide composition as the input. In 2009, Gao et al. [14] reconstructed the benchmark dataset for NRs and introduced the pseudo amino acid composition (PseAAC) [15] to represent the protein samples in hope to improve the prediction quality. As pioneering efforts in this area, the works by Bhasin and Raghava [13] and Gao et al. [14] did play a stimulating role in this area. However, the above two predictors have the following problems needed to be further addressed: (1) The benchmark datasets used to train the two predictors only covered four subfamilies, too narrow for the coverage scope. (2) There are many high homologous sequences included in their benchmark datasets because the cutoff threshold set by these authors to remove homologous sequences was 90%; a much more stringent threshold should be adopted to avoid homology bias. (3) The predictions by the two predictors were made under the assumption that the input query sequences are already known belonging to NRs; in other words, they cannot be used to identify whether a query protein as a NR or non-NR. (4) No web-server was provided [13] or the web-server provided by [14] is currently not working, and hence their methods cannot be easily used by the majority of experimental scientists to acquire the desired data for basic research and drug development.

To address the aforementioned four problems, recently a different predictor was proposed by extending the coverage scope from the four subfamilies of NRs as covered in [13], [14] to seven subfamilies [16]. The name of that predictor is called **NR-2L**, where 2L means that it is a two-level predictor. The 1^st^ level is for identifying query proteins as NRs or non-NRs, while the 2^nd^ level for identifying the NRs among their seven subfamilies.

In view of the importance of NRs to both basic research and drug development, the present study was initiated in an attempt to further improve the prediction quality of **NR-2L** by developing a new and more powerful predictor for identifying NRs and their subfamilies.

According to a recent review [17], to establish a really useful statistical predictor for a protein system, we need to consider the following procedures: (1) construct or select a high quality benchmark dataset to train and test the predictor; (2) formulate the protein samples with an effective mathematical expression that can truly reflect their intrinsic correlation with the target to be predicted; (3) introduce or develop a powerful algorithm (or engine) to operate the prediction; (4) properly perform cross-validation tests to objectively evaluate the anticipated accuracy of the predictor; (5) establish a user-friendly web-server for the predictor that is accessible to the public. Below, let us describe how to follow the above procedures to develop a new predictor that can further enhance the prediction quality in identifying NRs and their subfamilies.

## Materials and Methods

### 1. Benchmark Datasets

In this study, we selected the datasets from [16] as the benchmark dataset. The reason for doing so is because that the datasets constructed in [16] for establishing the predictor **NR-2L** are relatively more rigorous, and that it is also more convenient to compare our new predictor with **NR-2L** by using a same benchmark dataset. The benchmark dataset in [16] can be formulated as
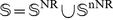
(1)where 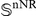 contains 500 non-NR protein sequences; while 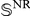 contains 159 protein sequences classified into the following seven subfamilies: (**1**) NR1: thyroid hormone like (thyroid hormone, retinoic acid, RAR-related orphan receptor, peroxisome proliferator activated, vitamin D3-like), (**2**) NR2: HNF4-like (hepatocyte nuclear factor 4, retinoic acid X, tailless-like, COUP-TF-like, USP), (**3**) NR3: estrogen like (estrogen, estrogen-related, glucocorticoid-like), (**4**) NR4: nerve growth factor IB-like (NGFI-B-like), (**5**) NR5: fushi tarazu-F1 like (fushi tarazu-F1 like), (**6**) NR6: germ cell nuclear factor like (germ cell nuclear factor), and (**7**) NR0: knirps like (knirps, knirps-related, embryonic gonad protein, ODR7, trithorax) and DAX like (DAX, SHP). For the dataset 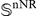, none of the proteins therein has 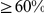 pairwise sequence identity to any other; for each of the seven subsets in 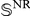, none of the proteins included has 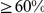 pairwise sequence identity to any other in a same subset. Listed in **Table 1** is a breakout of the proteins in the benchmark dataset used in the current study. The codes and sequences for the proteins in the benchmark dataset 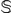 can be obtained from the Supporting Information S1 of [16] or directly downloaded from the website http://icpr.jci.edu.cn/bioinfo/NR2L/Supp.html.

**Table 1:** Breakdown of the benchmark dataset 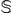 (cf. **Eq. 1**) used in this study.

Attribute	Dataset	Subfamily	Subset	Number
NR	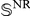	NR1	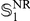	50
		NR2	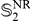	36
		NR3	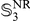	37
		NR4	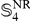	7
		NR5	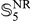	12
		NR6	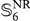	5
		NR0	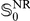	12
Non-NR	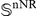	N/A	N/A	500

### 2. Protein Sequence Formulation

One of the keys in developing a method for identifying protein attributes is to formulate the protein samples with an effective mathematical expression that can truly reflect their intrinsic correlation with the target to be predicted [18]. However, it is by no means an easy job to realize this because this kind of correlation is usually deeply “buried” or “hidden” in piles of complicates sequences.

The most straightforward method to formulate the sample of a query protein 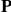 was just using its entire amino acid sequence, as can be generally described by
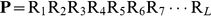
(2)where 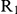 represents the 1^st^ residue of the protein 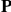, 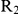 the 2^nd^ residue, …, 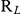 the 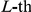 residue, and they each belong to one of the 20 native amino acids. In order to identify its attribute, the sequence-similarity-search-based tools, such as BLAST [19], [20], was utilized to search protein database for those proteins that have high sequence similarity to the query protein 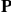. Subsequently, the attribute annotations of the proteins thus found were used to deduce the attribute for the query protein 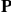. Unfortunately, this kind of straightforward sequential model, although quite intuitive and able to contain the entire information of a protein sequence, failed to work when the query protein 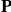 did not have significant sequence similarity to any attribute-known proteins.

Thus, various non-sequential or discrete models to formulate protein samples were proposed in hopes to establish some sort of correlation or cluster manner by which to enhance the prediction power.

Among the discrete models for a protein sample, the simplest one is its amino acid (AA) composition or AAC [21]. According to the AAC-discrete model, the protein 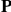 of **Eq. 4** can be formulated by [22]

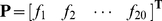
(3)where 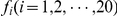 are the normalized occurrence frequencies of the 20 native amino acids in protein 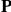, and 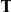 the transposing operator. Many methods for predicting various protein attributes were based on the AAC-discrete model (see, e.g., [21], [23], [24], [25], [26]). However, as we can see from **Eq. 3**, if using the ACC model to represent the protein 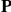, all its sequence-order effects would be lost, and hence the prediction quality might be considerably limited. To avoid completely losing the sequence-order information, instead of the simple amino acid composition (AAC), the pseudo amino acid composition (PseAAC) was proposed [15] to represent the protein samples.

The PseAAC approach has been widely used by investigators to predict various attributes of proteins, such as identifying bacterial virulent proteins [27], predicting homo-oligomeric proteins [28], identifying metalloproteinase family [29], predicting protein secondary structure content [30], predicting supersecondary structure [31], predicting protein structural classes [32], predicting enzyme family and sub-family classes [33], [34], [35], predicting protein subcellular location [36], [37], identifying cell wall lytic enzymes [38], identifying risk type of human papillomaviruses [39], predicting apoptosis protein subcellular location [40], [41], [42], [43], predicting outer membrane proteins [44], predicting subnuclear protein location [45], identifying bacterial secreted proteins [46], predicting protein submitochondria locations [47], [48], [49], predicting G-Protein-Coupled Receptor Classes [50], [51], predicting protein folding rates [52], predicting cyclin proteins [53], predicting GABA(A) receptor proteins [54], identifying the cofactors of oxidoreductases [55], identifying lipase types [56], identifying protease family [57], predicting Golgi protein types [58], among many others.

According to a recent review article [17], the general form of PseAAC for a protein 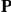 can be formulated as
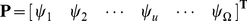
(4)where the subscript 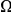 is an integer and its value as well as the components 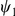, 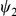, … will depend on how to extract the desired information from the amino acid sequence of 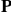.

Below, we are to use the “Physical-Chemical Property Matrix” and “Auto- and Cross- Covariance Transformation’ to define the 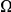 elements in **Eq. 4**.

#### 2.1. Physical-chemical property matrix

Each of the constituent amino acids in a protein has many physical-chemical properties. Therefore, a protein sequence can be encoded by a series of physical-chemical property values. In this study, the following ten physical-chemical (PC) properties were adopted: (1) PC^1^: hydrophobicity [59]; (2) PC^2^: hydrophilicity [60]; (3) PC^3^: side-chain mass (which can be obtained from any biochemistry text book), (4) PC^4^: pK1 (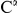-COOH [61]; (5) PC^5^: pK2 (NH3) [61]; (6) PC^6^: PI (25°C) [62]; (7) PC^7^: average buried volume; (8) PC^8^: molecular weight; (9) PC^9^: side chain volume; (10) PC^10^: mean polarity.

Thus, according to the ten PC properties, the protein 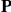 of **Eq. 2** can be formulated with a 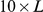 physical-chemical property matrix as given by
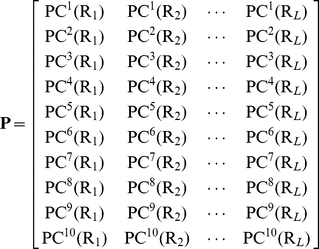
(5)where PC*^i^*(R*_j_*) is the value of PC*^i^* 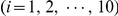 for residue R*_j_* 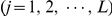.

Of the ten PC properties, the values for the first six can be directly obtained from the website http://www.csbio.sjtu.edu.cn/bioinf/PseAAC/PseAAReadme.htm, a part of the web-server **PseAAC** established for computing pseudo amino acid compositions of proteins according to their sequences [63]. The remainder can be obtained from **AAindex** (http://www.genome.jp/aaindex/), which is a database of numerical indices for various physicochemical and biochemical properties of amino acids and pairs of amino acids. All data in this database [64], [65] are derived from published literatures. Listed in **Table 2** are the values for the ten PC properties of the 20 amino acids, respectively. However, before submitting them into **Eq. 5**, all the data in **Table 2** were subject to a standard conversion through the following equation [66]:
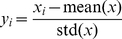
(6)where 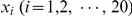 stands for the original score of the *i*th amino acid, 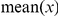 for the mean score of the 20 amino acids, and 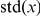 for the corresponding standard deviation. The converted values obtained via **Eq. 6** will have a zero mean value over the 20 amino acids, and will remain unchanged if they go through the same conversion procedure again [66].

**Table 2:** List of the values of the ten physical-chemical properties for each of the 20 native amino acids.

Amino acid	PC^1^	PC^2^	PC^3^	PC^4^	PC^5^	PC^6^	PC^7^	PC^8^	PC^9^	PC^10^
A	0.62	−0.5	15	2.35	9.87	6.11	91.50	89.09	27.5	−0.06
C	0.29	−1.00	47	1.71	10.78	5.02	117.7	121.2	44.6	1.36
D	−0.90	3.00	59	1.88	9.60	2.98	124.5	133.1	40.0	−0.80
E	−0.74	3.00	73	2.19	9.67	3.08	155.1	147.1	62.0	−0.77
F	1.19	−2.50	91	2.58	9.24	5.91	203.4	165.2	115.5	1.27
G	0.48	0.00	1	2.34	9.60	6.06	66.40	75.07	0.0	−0.41
H	−0.40	−0.50	82	1.78	8.97	7.64	167.3	155.2	79.0	0.49
I	1.38	−1.80	57	2.32	9.76	6.04	168.8	131.2	93.5	1.31
K	−1.50	3.00	73	2.20	8.90	9.47	171.3	146.2	100.0	−1.18
L	1.06	−1.80	57	2.36	9.60	6.04	167.9	131.2	93.5	1.21
M	0.64	−1.30	75	2.28	9.21	5.74	170.8	149.2	94.1	1.27
N	−0.78	0.20	58	2.18	9.09	10.76	135.2	132.1	58.7	−0.48
P	0.12	0.00	42	1.99	10.60	6.30	129.3	115.1	41.9	0.00
Q	−0.85	0.20	72	2.17	9.13	5.65	161.1	146.2	80.7	−0.73
R	−2.53	3.00	101	2.18	9.09	10.76	202.0	174.2	105	−0.84
S	−0.18	0.30	31	2.21	9.15	5.68	99.10	105.1	29.3	−0.50
T	−0.05	−0.40	45	2.15	9.12	5.60	122.1	119.1	51.3	−0.27
V	1.08	−1.50	43	2.29	9.74	6.02	141.7	117.2	71.5	1.09
W	0.81	−3.40	130	2.38	9.39	5.88	237.6	204.2	145.5	0.88
Y	0.26	−2.30	107	2.20	9.11	5.63	203.6	181.2	117.3	0.33

Thus, given a protein with 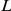 amino acids, it can be expressed as a 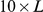 numerical matrix via the ten physical-chemical properties as given in Eq. 5. Such a matrix is called the physical-chemical property matrix or PC matrix, for protein 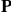. It is assumed that those NRs that belong to a same type should have a similar PC matrix, or vice versa.

#### 2.2. Auto-covariance and cross-covariance

**Figure 1:**
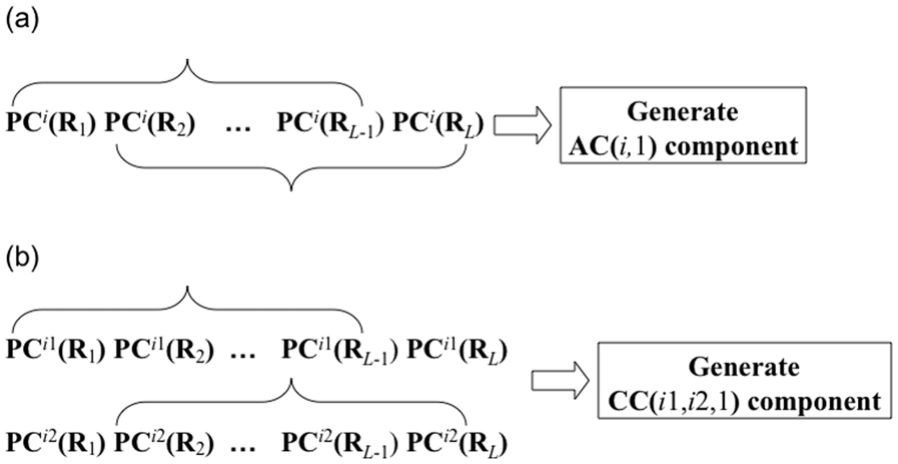
An illustration to show two types of covariance. (**a**) The auto-covariance refers to the coupling between two subsequences from a same sequence when they are separated by 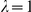 unit. (**b**) The cross-covariance refers to the coupling between two subsequences from two different sequences as indicated by two open curly braces.

In statistics, the auto-covariance is the covariance of a stochastic process against a parameter-shifted version of itself (**Fig. 1a**), while the cross-covariance is used to refer to the covariance between two random vectors (**Fig. 1b**). Here, let us use the two concepts of covariance to transform the matrix of **Eq. 5** to a length-fixed feature vector, as described below.

According to the concept of auto-covariance (AC), the correlation of the same PC property between two subsequences separated by 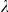 amino acids can be formulated as
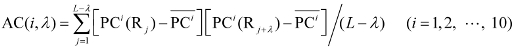
(7)where 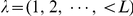 [15] and 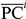 represents the mean value of the *i*th horizontal line in **Eq. 5**, as given by
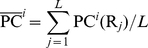
(8)As we can see from **Eq. 7**, using auto-covariance on the physical-chemical property matrix of **Eq. 5**, we can generate 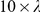 auto-covariance components.

On the other hand, according to the concept of cross-covariance (CC), the correlation between two subsequences with each belonging to a different PC property can be formulated by
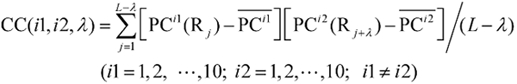
(9)Hence, using cross-covariance on the physical-chemical property matrix of **Eq. 5**, we can generate 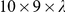 cross-covariance components.

Accordingly, a total of 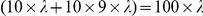 components can thus be generated from **Eq. 5**. However, it was indicated by preliminary computations and analyses that when 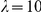, better results would be obtained. Thus, in this study the PseAAC for protein 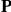 is expressed as
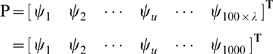
(10)where 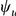 is the 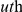 components generated by operating the above auto-covariance and cross-covariance on the physical-chemical property matrix of **Eq. 5**.

#### 2.3. Support vector machines

Support vector machines (SVMs) are a set of related supervised learning methods that are usually used to analyze data and recognize patterns. The original SVM algorithm was proposed by Vapnik [67] and the current standard incarnation (soft margin) was proposed by Cortes and Vapnik [68]. When used in the current study, its mathematical principles can be briefly described as follows.

Given a set of 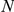 samples, i.e. a series of input vectors
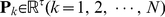
(11)where 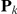 can be regarded as the 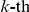 protein sample or vector as formulated by **Eq. 10**, and 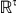 is an Euclidean space with 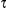 dimensions. For the current case, 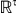 is actually a PseAAC space with 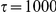 (cf. **Eq. 10**). The SVM algorithm performs a mapping of the vectors of proteins in the training dataset from the space 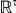 into a higher dimensional space 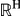 by a kernel function and finds an optimal separating hyperplane, which maximizes the margin between the hyperplane and the nearest data points of each class in the space 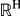. Different kernel functions define different SVMs. In principle, SVM is a two-class classifier. With the recent improvements, the SVM can now be directly used to cope with multi-class classification problem via the one-against-all or pairwise approach. For the detailed mathematical formulations, see Eqs. 3–18 in [69], where instead of the 1000-D PseAAC space, a protein sample was defined in the 2005-D FunD (functional domain) composition space.

SVM has been widely used to classify various attributes of proteins according to their sequences information (see, e.g., [33], [43], [69], [70], [71], [72], [73], [74]). In this study, the LIBSVM package [75] was used as an implementation of SVM, which can be downloaded from http://www.csie.ntu.edu.tw/~cjlin/libsvm/, the popular radial basis function (RBF) was taken as the kernel function. For the current SVM classifier, there were two unknown parameter: penalty parameter 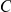 and kernel parameter 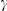. The method of how to determine the two parameters will be discussed later.

**Figure 2:**
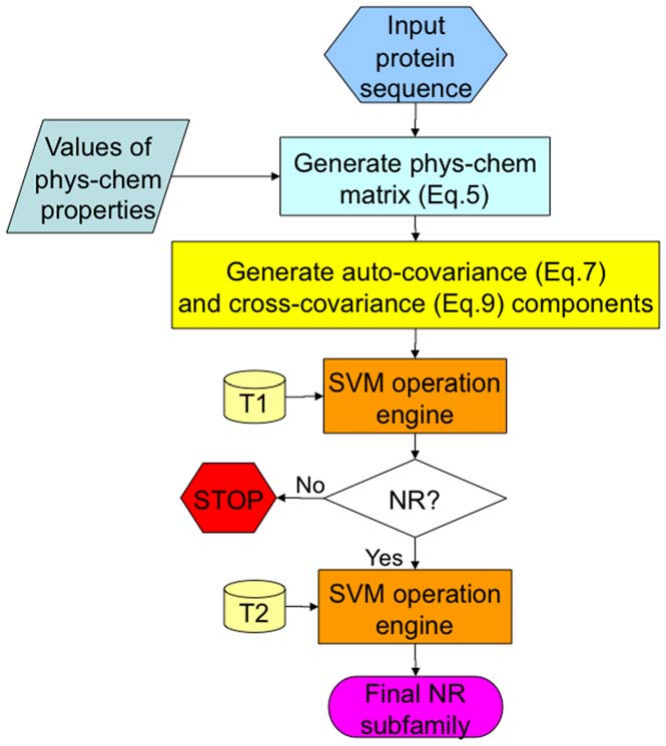
A flowchart to show the prediction process of iNR-PhysChem. T1 represents the benchmark dataset from [16] for training the 1^st^-level prediction; T2 represents the benchmark dataset from [16] for training the 2^nd^-level prediction. See the text for further explanation.

The predictor established via the aforementioned procedures is called **iNR-PhysChem**, where the character “i” stands for “identifying”, “NR” for “nuclear receptors and their subfamilies”, and “PhysChem” for “using physical-chemical property features”. To provide an intuitive overall picture, a flowchart is provided in **Fig. 2** to illustrate the process of how **iNR-PhysChem** works in identifying nuclear receptors and their subfamilies.

#### 2.4. Performance metrics

**Figure 3:**
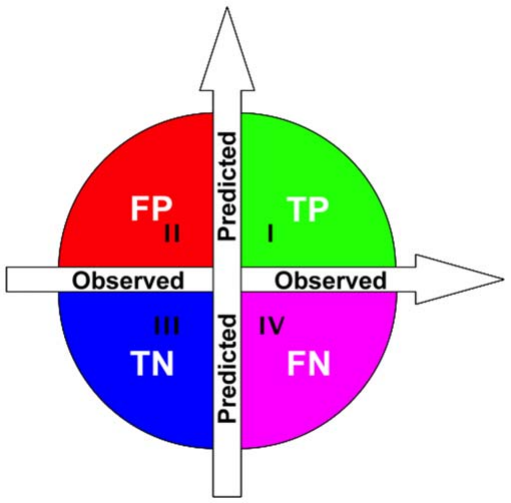
An illustration to show the predicted results fallen into four different quadrants. (I) TP, the true positive quadrant (green) for correct prediction of positive dataset, (II) FP, the false positive quadrant (red) for incorrect prediction of negative dataset; (III) TN, the true negative quadrant (blue) for correct prediction of negative dataset; and (IV) FN, the false negative quadrant (pink) for incorrect prediction of positive dataset.

The performance of the predictor is evaluated by the overall accuracy, which is the most commonly used metric for assessing the global performance of a multi-class problem. The overall accuracy (*ACC*) is defined as the ratio of correctly predicted samples to all tested samples:
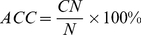
(12)where 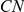 is the number of proteins whose attribute have been correctly identified and 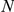 the total number of proteins in the benchmark dataset. Also, to examine the stability of the pretictor, the Matthew's correlation coefficient (*MCC*) is computed according to the following formulation:
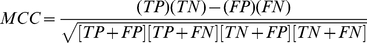
(13)where *TP* represents the true positive; *TN*, the true negative; *FP*, the false positive; and *FN*, the false negative (**Fig. 3**).

#### 2.5. Web-server guide

The mathematic equations presented above are just for the integrity in developing the **iNR-PhysChem** predictor. For those who are interested in only using **iNR-PhysChem**, a web-server has been established. Below, let us give a step-by-step guide on how to use it to get the desired results without the need to follow the complicated mathematic equations at all.

#### Step 1

**Figure 4:**
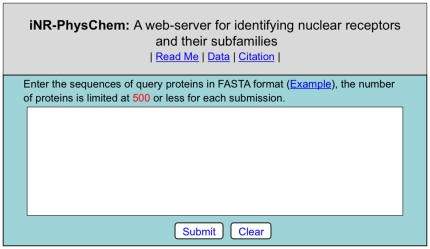
A semi-screenshot to see the top page of iNR-PhysChem. The web-server is at either http://www.jci-bioinfo.cn/iNR-PhysChem or http://icpr.jci.edu.cn/bioinfo/iNR-PhysChem.

Open the web server at either http://www.jci-bioinfo.cn/iNR-PhysChem or http://icpr.jci.edu.cn/bioinfo/iNR-PhysChem, and you will see the top page of the predictor on your computer screen, as shown in **Fig. 4**. Click on the Read Me button to see a brief introduction about the **iNR-PhysChem** predictor, and its anticipated accuracy.

#### Step 2

Either type or copy/paste the query protein sequence into the input box at the center of **Fig. 4**. The input sequence should be in the FASTA format. A sequence in FASTA format consists of a single initial line beginning with a greater-than symbol (“>”) in the first column, followed by lines of sequence data. The words right after the “>” symbol in the single initial line are optional and only used for the purpose of identification and description. The sequence ends if another line starting with a “>” appears; this indicates the start of another sequence. Example sequences in FASTA format can be seen by clicking on the Example button right above the input box. The maximum number of query proteins allowed for each submission is 500.

#### Step 3

Click on the Submit button to see the predicted result. For example, if you use the two query protein sequences in the Example window as the input, after clicking the Submit button, you will see from your computer screen that the 1^st^ query protein (THB2_RAT) is a “**NR**” belonging to the subfamily of “**NR1 (Thyroid hormone like)**”, and that the 2^nd^ query protein (E1FMC1_LOALO) is a “**non-NR**”. All these results are fully consistent with the experimental observations. It only took a few seconds to get the above results. If the input contains 500 query protein sequences, the job will be finished in less than 2 minutes.

#### Step 4

Click on the Data button to download the benchmark datasets used to train and test the **iNR-PhysChem** predictor.

#### Step 5

Click on the Citation button to find the relevant paper that documents the development of the **iNR-PhysChem** predictor.

## Results and Discussion

In statistical prediction, the following three cross-validation methods are often used to examine a predictor for its effectiveness in practical application: independent dataset test, K-fold (such as 5-fold, 7-fold, or 10-fold) subsampling test, and jackknife test [76]. However, as elucidated by [77] and demonstrated by Eqs. 28–32 of [17], among the three cross-validation methods, the jackknife test is deemed the least arbitrary that can always yield a unique result for a given benchmark dataset, and hence has been increasingly used and widely recognized by investigators to examine the accuracy of various predictors (see, e.g., [37], [39], [50], [53], [78], [79]). Therefore, in this study the jackknife cross-validation was adopted to calculate the success prediction rates as well.

**Figure 5:**
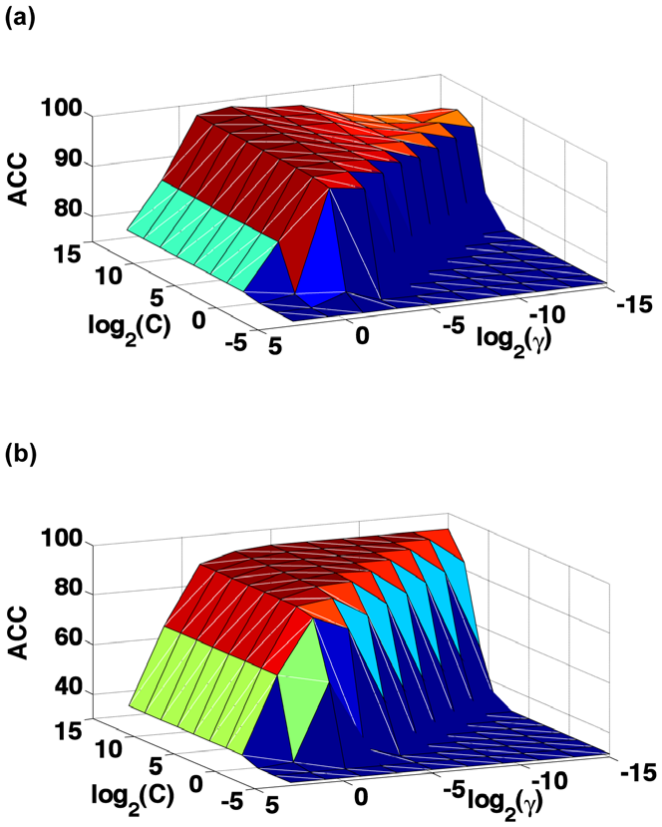
The 3D graph to show the success rates by the 5-fold cross-validation with different values of *C* and 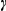 in the SVM engine. (**a**) The results obtained for the 1^st^-level prediction. (b) The results obtained for the 2^nd^-level prediction.

However, for a system involved with two uncertain parameters (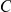 and 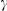), it will need a lot of computational times to find their optimal values. Therefore, as a fist step, let us determine the values of 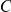 and 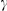 for the current SVM operation engine just by optimizing the overall 5-fold cross-validation success rate thru a 2-D grid search (**Fig. 5**). The values thus obtained for the two parameters are given by
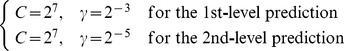
(14)where the 1^st^-level prediction is for identifying a query protein as NR or non-NR; while the 2^nd^-level prediction is for identifying a NR among its seven subfamilies (cf. **Table 1**).

Subsequently, using the parameters values of **Eq. 14** for the SVM operation engine, the jackknife tests were performed on the benchmark dataset 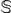 (cf. **Eq. 1**).

The results thus obtained in identifying proteins as NRs or non-NRs are given in **Table 3**; while those in identifying NRs among their seven subfamilies are given in **Table 4**. For facilitating comparison, the corresponding results obtained by the predictor **NR-2L** [16] are also listed in the two tables.

**Table 3:** Comparison of the success rates and *MCC* values obtained by the current iNR-PhysChem and NR-2L [16] in identifying NRs and non-NRs by the jackknife test on the benchmark dataset 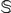 (cf. **Eq. 1**).

Attribute	iNR-PhysChem		NR-2L	
	*ACC*	*MCC*	*ACC*	*MCC*
NR	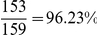	0.95	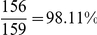	0.83
Non-NR	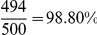	0.95	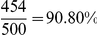	0.83
Overall	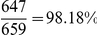	0.96	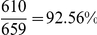	0.85

**Table 4:** Comparison of the success rates and *MCC* values obtained by the current **iNR-PhysChem** and **NR-2L** [16] in identifying the subfamilies of NRs by the jackknife test on the benchmark dataset 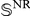 (cf. **Eq. 1**).

NR subfamily	iNR-PhysChem		NR-2L	
	*ACC*	*MCC*	*ACC*	*MCC*
NR1	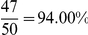	0.87	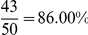	0.88
NR2	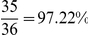	0.93	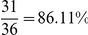	0.85
NR3	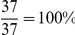	0.95	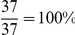	0.86
NR4	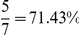	0.84	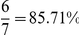	0.70
NR5	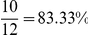	0.91	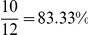	0.86
NR6	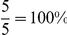	1.00	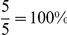	1.00
NR0	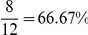	0.81	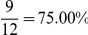	0.86
Overall	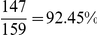	0.91	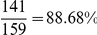	0.87

As we can see from **Table 3**, the overall jackknife success rate in identifying NRs and non-NRs by the current **iNR-PhysChem** is 98.16%, which is obviously higher than the corresponding rate by the **NR-2L** predictor [16]. Meanwhile, the overall *MCC* by **iNR-PhysChem** is 0.96, which is also more close to 1 than that by the **NR-2L** predictor [16]. Also, it can be seen from **Table 4**, the overall jackknife success rate in identifying NRs among their seven subfamilies and the overall *MCC* by the current **iNR-PhysChem** are 92.45% and 0.91, respectively, which are also higher than the corresponding rates by the **NR-2L** predictor [16]. All these results indicate that the current **iNR-PhysChem** is superior to **NR-2L** [16] not only in achieving higher success rates, but also in getting more stable predicted results.

The higher success rates with more stability indicate that it is a promising strategy to use the physical-chemical matrix to investigate the attributes of proteins, and that it can catch the essential features of NRs by representing their sequence samples with PseAAC consisting of the components derived from their physical-chemical matrix via the auto-covariance and cross-covariance transformation.

It is anticipated that **iNR-PhysChem** may become a useful high throughput tool for both basic research and drug development.

Finally, people might be interested to know how to rank the impacts of the ten amino acid properties (cf. **Eq. 5**) for their roles in identifying the NRs and their subfamilies. To address this problem, a leave-one-out test was performed for each of the ten amino acid properties. The property would be deemed having the most significant impact if the overall success rate dropped down the most after excluding it from the ten properties. It was observed that for the 1^st^- level prediction (i.e., in identifying query proteins as NRs or non-NRs), their impacts were ranked as
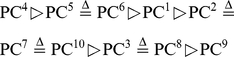
(15)where the symbol 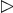 means “greater than in impact”, and the symbol 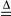 means “equal to in impact”. For the 2^nd^-level prediction (i.e., in identifying the NRs among their seven subfamilies), the impacts of the ten amino acid properties were ranked as
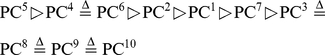
(16)In other words, pK1 had the highest impact in identifying query proteins as NRs or non-NRs, followed by pK2 and PI, and so forth (cf. Section 2.1 of Materials and Methods); while pK2 had the highest impact in identifying NRs among their seven subfamilies, followed by pK1 and PI, and so forth.

## References

[1] Evans RM (1988). The steroid and thyroid hormone receptor superfamily.. Science.

[2] Olefsky JM (2001). Nuclear Receptor Minireview Series.. Journal of Biological Chemistry.

[3] Altucci L, Gronemeyer H (2001). Nuclear receptors in cell life and death.. Trends in Endocrinology and Metabolism.

[4] Florence H, Gerrit V, Fred EC (2001). Collecting and harvesting biological data: the GPCRDB and NucleaRDB information systems.. Nucleic Acids Research.

[5] Mangelsdorf DJ, Thummel C, Beato M, Herrlich P, Schultz G (1995). The nuclear receptor superfamily: The second decade.. Cell.

[6] Laudet V, Gronemeyer H (2002). The nuclear receptors factsbook.

[7] Novac N, Heinzel T (2004). Nuclear receptors: overview and classification.. Current drug targets Inflammation and allergy.

[8] Gronemeyer H, Laudet V (1995). Transcription factors 3: nuclear receptors.. Protein Profile.

[9] Kumar R, Thompson EB (1999). The structure of the nuclear hormone receptors.. Steroids.

[10] Robinson-Rechavi M, Garcia HE, Laudet V (2003). The nuclear receptor superfamily.. J Cell Sci.

[11] Nuclear-Receptors-Committee (1999). A Unified Nomenclature System for the Nuclear Receptor Superfamily.. Cell.

[12] Laudet V (1997). Evolution of the nuclear receptor superfamily: early diversification from an ancestral orphan receptor.. J Mol Endocrinol.

[13] Bhasin M, Raghava GP (2004). ESLpred: SVM-based method for subcellular localization of eukaryotic proteins using dipeptide composition and PSI-BLAST.. Nucleic Acids Res.

[14] Gao Q-B, Jin Z-C, Ye X-F, Wu C, He J (2009). Prediction of nuclear receptors with optimal pseudo amino acid composition.. Analytical Biochemistry.

[15] Chou KC (2001). Prediction of protein cellular attributes using pseudo amino acid composition.. PROTEINS: Structure, Function, and Genetics (Erratum: ibid, 2001, Vol44, 60).

[16] Wang P, Xiao X, Chou KC (2011). NR-2L: A Two-Level Predictor for Identifying Nuclear Receptor Subfamilies Based on Sequence-Derived Features.. PLoS ONE.

[17] Chou KC (2011). Some remarks on protein attribute prediction and pseudo amino acid composition (50th Anniversary Year Review).. Journal of Theoretical Biology.

[18] Chou KC (2009). Pseudo amino acid composition and its applications in bioinformatics, proteomics and system biology.. Current Proteomics.

[19] Altschul SF, Suhai S (1997). Evaluating the statistical significance of multiple distinct local alignments.. Theoretical and Computational Methods in Genome Research.

[20] Wootton JC, Federhen S (1993). Statistics of local complexity in amino acid sequences and sequence databases.. Comput Chem.

[21] Nakashima H, Nishikawa K, Ooi T (1986). The folding type of a protein is relevant to the amino acid composition.. J Biochem.

[22] Chou KC, Zhang CT (1994). Predicting protein folding types by distance functions that make allowances for amino acid interactions.. Journal of Biological Chemistry.

[23] Zhou GP (1998). An intriguing controversy over protein structural class prediction.. Journal of Protein Chemistry.

[24] Chou KC, Elrod DW (1999). Protein subcellular location prediction.. Protein Engineering.

[25] Zhou GP, Assa-Munt N (2001). Some insights into protein structural class prediction.. PROTEINS: Structure, Function, and Genetics.

[26] Zhou GP, Doctor K (2003). Subcellular location prediction of apoptosis proteins.. PROTEINS: Structure, Function, and Genetics.

[27] Nanni L, Lumini A, Gupta D, Garg A (2011). Identifying Bacterial Virulent Proteins by Fusing a Set of Classifiers Based on Variants of Chou's Pseudo Amino Acid Composition and on Evolutionary Information..

[28] Qiu JD, Suo SB, Sun XY, Shi SP, Liang RP (2011). OligoPred: A web-server for predicting homo-oligomeric proteins by incorporating discrete wavelet transform into Chou's pseudo amino acid composition.. J Mol Graph Model.

[29] Mohammad Beigi M, Behjati M, Mohabatkar H (2011). Prediction of metalloproteinase family based on the concept of Chou's pseudo amino acid composition using a machine learning approach.. Journal of Structural and Functional Genomics.

[30] Chen C, Chen L, Zou X, Cai P (2009). Prediction of protein secondary structure content by using the concept of Chou's pseudo amino acid composition and support vector machine.. Protein & Peptide Letters.

[31] Zou D, He Z, He J, Xia Y (2011). Supersecondary structure prediction using Chou's pseudo amino acid composition.. Journal of Computational Chemistry.

[32] Sahu SS, Panda G (2010). A novel feature representation method based on Chou's pseudo amino acid composition for protein structural class prediction.. Computational Biology and Chemistry.

[33] Qiu JD, Huang JH, Shi SP, Liang RP (2010). Using the concept of Chou's pseudo amino acid composition to predict enzyme family classes: an approach with support vector machine based on discrete wavelet transform.. Protein & Peptide Letters.

[34] Zhou XB, Chen C, Li ZC, Zou XY (2007). Using Chou's amphiphilic pseudo-amino acid composition and support vector machine for prediction of enzyme subfamily classes.. Journal of Theoretical Biology.

[35] Wang YC, Wang XB, Yang ZX, Deng NY (2010). Prediction of enzyme subfamily class via pseudo amino acid composition by incorporating the conjoint triad feature.. Protein & Peptide Letters.

[36] Li FM, Li QZ (2008). Predicting protein subcellular location using Chou's pseudo amino acid composition and improved hybrid approach.. Protein & Peptide Letters.

[37] Chou KC, Wu ZC, Xiao X (2012). iLoc-Hum: Using accumulation-label scale to predict subcellular locations of human proteins with both single and multiple sites.. Molecular Biosystems.

[38] Ding H, Luo L, Lin H (2009). Prediction of cell wall lytic enzymes using Chou's amphiphilic pseudo amino acid composition.. Protein & Peptide Letters.

[39] Esmaeili M, Mohabatkar H, Mohsenzadeh S (2010). Using the concept of Chou's pseudo amino acid composition for risk type prediction of human papillomaviruses.. Journal of Theoretical Biology.

[40] Ding YS, Zhang TL (2008). Using Chou's pseudo amino acid composition to predict subcellular localization of apoptosis proteins: an approach with immune genetic algorithm-based ensemble classifier.. Pattern Recognition Letters.

[41] Lin H, Wang H, Ding H, Chen YL, Li QZ (2009). Prediction of Subcellular Localization of Apoptosis Protein Using Chou's Pseudo Amino Acid Composition.. Acta Biotheoretica.

[42] Jiang X, Wei R, Zhang TL, Gu Q (2008). Using the concept of Chou's pseudo amino acid composition to predict apoptosis proteins subcellular location: an approach by approximate entropy.. Protein & Peptide Letters.

[43] Kandaswamy KK, Pugalenthi G, Moller S, Hartmann E, Kalies KU (2010). Prediction of Apoptosis Protein Locations with Genetic Algorithms and Support Vector Machines Through a New Mode of Pseudo Amino Acid Composition.. Protein and Peptide Letters.

[44] Lin H (2008). The modified Mahalanobis discriminant for predicting outer membrane proteins by using Chou's pseudo amino acid composition.. Journal of Theoretical Biology.

[45] Jiang X, Wei R, Zhao Y, Zhang T (2008). Using Chou's pseudo amino acid composition based on approximate entropy and an ensemble of AdaBoost classifiers to predict protein subnuclear location.. Amino Acids.

[46] Yu L, Guo Y, Li Y, Li G, Li M (2010). SecretP: Identifying bacterial secreted proteins by fusing new features into Chou's pseudo-amino acid composition.. Journal of Theoretical Biology.

[47] Lin H, Ding H, Feng-Biao Guo FB, Zhang AY, Huang J (2008). Predicting subcellular localization of mycobacterial proteins by using Chou's pseudo amino acid composition.. Protein & Peptide Letters.

[48] Zeng YH, Guo YZ, Xiao RQ, Yang L, Yu LZ (2009). Using the augmented Chou's pseudo amino acid composition for predicting protein submitochondria locations based on auto covariance approach.. Journal of Theoretical Biology.

[49] Nanni L, Lumini A (2008). Genetic programming for creating Chou's pseudo amino acid based features for submitochondria localization.. Amino Acids.

[50] Gu Q, Ding YS, Zhang TL (2010). Prediction of G-Protein-Coupled Receptor Classes in Low Homology Using Chou's Pseudo Amino Acid Composition with Approximate Entropy and Hydrophobicity Patterns.. Protein & Peptide Letters.

[51] Qiu JD, Huang JH, Liang RP, Lu XQ (2009). Prediction of G-protein-coupled receptor classes based on the concept of Chou's pseudo amino acid composition: an approach from discrete wavelet transform.. Analytical Biochemistry.

[52] Guo J, Rao N, Liu G, Yang Y, Wang G (2011). Predicting protein folding rates using the concept of Chou's pseudo amino acid composition.. Journal of Computational Chemistry.

[53] Mohabatkar H (2010). Prediction of cyclin proteins using Chou's pseudo amino acid composition.. Protein & Peptide Letters.

[54] Mohabatkar H, Mohammad Beigi M, Esmaeili A (2011). Prediction of GABA(A) receptor proteins using the concept of Chou's pseudo-amino acid composition and support vector machine.. Journal of Theoretical Biology.

[55] Zhang GY, Fang BS (2008). Predicting the cofactors of oxidoreductases based on amino acid composition distribution and Chou's amphiphilic pseudo amino acid composition.. Journal of Theoretical Biology.

[56] Zhang GY, Li HC, Gao JQ, Fang BS (2008). Predicting lipase types by improved Chou's pseudo-amino acid composition.. Protein & Peptide Letters.

[57] Hu L, Zheng L, Wang Z, Li B, Liu L (2011). Using pseudo amino Acid composition to predict protease families by incorporating a series of protein biological features.. Protein and Peptide Letters.

[58] Ding H, Liu L, Guo FB, Huang J, Lin H (2011). Identify Golgi protein types with modified mahalanobis discriminant algorithm and pseudo amino acid composition.. Protein & Peptide Letters.

[59] Tanford C (1962). Contribution of hydrophobic interactions to the stability of the globular conformation of proteins.. J Am Chem Soc.

[60] Hopp TP, Woods KR (1981). Prediction of protein antigenic determinants from amino acid sequences.. Proc Natl Acad Sci USA.

[61] Robert CW (1985). CRC Handbook of Chemistry and Physics, 66th edition.

[62] Dawson RMC, Elliott DC, Elliott WH, Jones KM (1986). Data for Biochemical Research 3rd edition.

[63] Shen HB, Chou KC (2008). PseAAC: a flexible web-server for generating various kinds of protein pseudo amino acid composition.. Analytical Biochemistry.

[64] Kawashima S, Ogata H, Kanehisa M (1999). AAindex: Amino Acid Index Database.. Nucleic Acids Research.

[65] Kawashima S, Pokarowski P, Pokarowska M, Kolinski A, Katayama T (2008). AAindex: amino acid index database, progress report 2008.. Nucleic Acids Research.

[66] Chou KC, Shen HB (2007). Review: Recent progresses in protein subcellular location prediction.. Analytical Biochemistry.

[67] Vapnik VN (1995). The Nature of Statistical Learning Theory.

[68] Cortes C, Vapnik V (1995). Support vector networks. Machine Learning.. Machine Learning.

[69] Chou KC, Cai YD (2002). Using functional domain composition and support vector machines for prediction of protein subcellular location.. Journal of Biological Chemistry.

[70] Cai YD, Zhou GP, Chou KC (2003). Support vector machines for predicting membrane protein types by using functional domain composition.. Biophysical Journal.

[71] Li D, Jiang Z, Yu W, Du L (2010). Predicting Caspase Substrate Cleavage Sites Based on a Hybrid SVM-PSSM Method.. Protein and Peptide Letters.

[72] Li YX, Shao YH, Deng NY (2011). Improved Prediction of Palmitoylation Sites Using PWMs and SVM.. Protein & Peptide Letters.

[73] Bhasin M, Raghava GPS (2004). Classification of Nuclear Receptors Based on Amino Acid Composition and Dipeptide Composition.. Journal of Biological Chemistry.

[74] Ding YS, Zhang TL, Chou KC (2007). Prediction of protein structure classes with pseudo amino acid composition and fuzzy support vector machine network.. Protein & Peptide Letters.

[75] Chang C-C, Lin C-J (2001). LIBSVM: a library for support vector machines.. http://www.csie.ntu.edu.tw/~cjlin/libsvm.

[76] Chou KC, Zhang CT (1995). Review: Prediction of protein structural classes.. Critical Reviews in Biochemistry and Molecular Biology.

[77] Chou KC, Shen HB (2008). Cell-PLoc: A package of Web servers for predicting subcellular localization of proteins in various organisms (updated version: Cell-PLoc 2.0: An improved package of web-servers for predicting subcellular localization of proteins in various organisms, Natural Science, 2010, 2, 1090–1103).. Nature Protocols.

[78] Georgiou DN, Karakasidis TE, Nieto JJ, Torres A (2009). Use of fuzzy clustering technique and matrices to classify amino acids and its impact to Chou's pseudo amino acid composition.. Journal of Theoretical Biology.

[79] Wu ZC, Xiao X, Chou KC (2011). iLoc-Plant: a multi-label classifier for predicting the subcellular localization of plant proteins with both single and multiple sitesw.. Molecular BioSystems.

